# 2D:4D ratio as a predictor for malocclusion among Saudi children

**DOI:** 10.6026/973206300210100

**Published:** 2025-01-31

**Authors:** Ahmed Ali Alfawzan, Mohammed Ali Habibullah, Nada Mohammed Aloufi, Mohamed Tharwat Salama

**Affiliations:** 1 Department of Orthodontic and Pediatric Dentistry, College of Dentistry, Qassim University, Kingdom of Saudi Arabia

**Keywords:** Biological marker, 2D:4D ratio, children, hormonal fingerprint and malocclusion

## Abstract

2D:4D ratio is the ratio of the lengths of the index and ring fingers. It is a potential indicator of prenatal hormonal exposure, reflecting androgen sensitivity and it may influence skeletal and dental growth. Therefore, it is of interest to explore the correlation between the 2D:4D ratio and malocclusion types using Angle's classification of malocclusion in 410 male students aged 12-15 years from Alrass City, Saudi Arabia. Statistical analysis using Chi-Square tests revealed no considerable association between the 2D:4D ratio and malocclusion type (p=0.904). This data suggest that the ratio does not serve as a reliable predictor highlighting the complexity of factors influencing malocclusion and the need for further studies to better interpret hormonal, genetic and environmental contributions to craniofacial growth.

## Background:

The role of genetics in craniofacial growth is a subject of interest for both the orthodontists and multiple other specialties. Controversies about the heredity of malocclusion traits have existed for many years. Some researchers highlighted the role of genetics in etiology of skeletal and dental malocclusion [1, 2]. However, studies on siblings and identical twins showed that both environmental and genetic factors have an impact on the development of occlusion with environmental factors predominating [3]. Hormonal fingerprint (2D:4D ratio) is the ratio of 2nd and 4th digit length. 2D:4D ratio has been used in medicine as a risk marker for diagnoses and correlating medical conditions in infancy. This usage is aided by the fact that like finger prints, the 2D:4D ratio is consistent and stable [4, 5]. The Hormonal fingerprint is sexually determined with men exhibiting a lower ratio than women and reflects the androgen sensitivity rather than concentration [6]. The use of Hormonal fingerprint is helpful for assessing body and behavior and prediction of malocclusion and caries risk in subjects [7, 8]. Therefore, it is of interest to assess the association between hormonal fingerprint and different types of malocclusion among Saudi children.

## Materials and Methods:

This cross-sectional research was conducted in Alrass city, Qassim, Saudi Arabia. Stratified cluster random sampling technique was used to derive the sample. There are 28 intermediate schools in Alrass and 10 schools (2 schools each from north, south, east, west and Central district of Alrass) were included. A sample of 323 male students was measured based on a 95% confidence level and a 5% margin of error, but a total of 410 male students aged 12-15 years was included in the study for practical reasons. Students who were eligible based on the inclusion and exclusion criteria and provided informed consent were included in the research. The study was approved by Ethical Clearance Committee of our institution.

## Inclusion criteria:

Healthy children who provided informed consent and have complete permanent dentition except third molars.

## Exclusion criteria:

Students with history of previous orthodontic treatment, history of trauma or presence of facial syndromes, hormonal imbalances, grossly carious teeth, missing permanent tooth, retained primary tooth, functional shift and apparent defect in the hand were excluded from this study.

The age details of students were derived from school records. All clinical examinations were performed by a single trained examiner who is a Saudi board-certified orthodontist. Examiner Calibration was achieved before the commencement of the study by examining a preselected group of 25 children twice at 2-day intervals. The kappa score was found to be above 0.87. An Electronic Digital Caliper (Model no.LL004B, manufactured by Guangzhou Juanjuan Electronic Technology Co., China) was used to calculate the length of the index and ring digit from the tip of the digit to the proximal creases of the digit on the ventral surface of the right hand.

## Calculation of 2D:4D Ratio:

The length of the index (2D) and Ring finger (4D) were evaluated from the proximal crease of the digit to the tip using a digital caliper device (Figure 1 - see PDF). The use of the digital caliper eliminated any error in the reading of the measurements. The digit ratio was deliberated by dividing the length of the index digit by the length of the ring digit using excels Excel-formulated function.

The molar relationship was assessed in centric occlusion. The sample is divided into three groups based on Angle's classification of malocclusion [9] (Class I, Class II and Class III). A single trained person assisted with documentation throughout the study. The Pearson's correlation test, t-test and Chi-Square test were performed using SPSS version 22.0; the difference was considered as statistically significant when P value < 0.05.

## Results:

**Figure 2:**
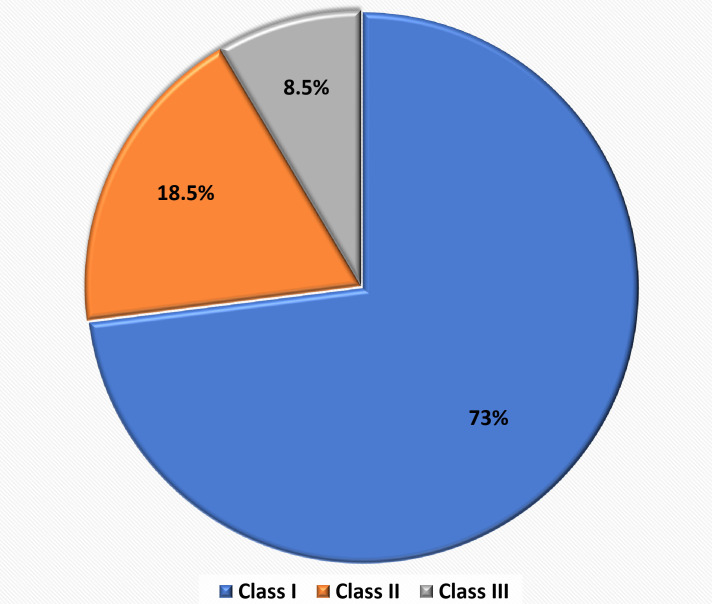
Prevalence of different molar relationship in the sample

**Figure 3:**
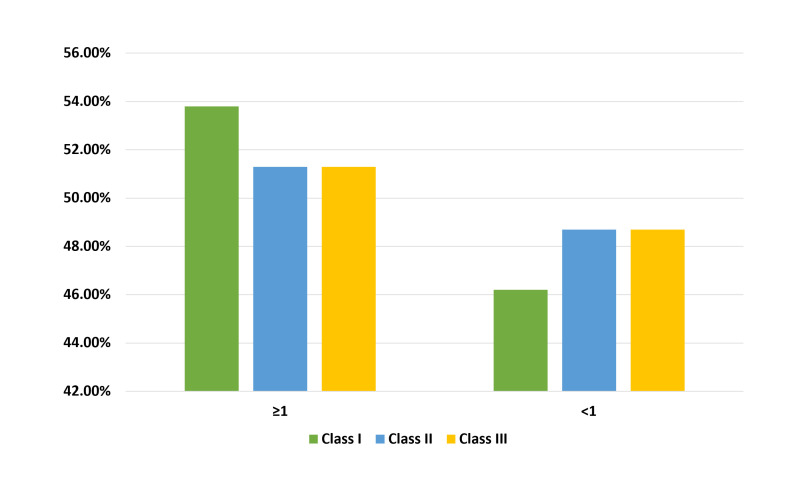
Distribution of the 2D:4D ration across the different malocclusion

A total of 410 male students aged 12 to 15 years from 10 schools in Alrass City, Saudi Arabia, participated in this study. The descriptive characteristics of the research population are provided in Table 1. The average age of the students was 13.00 years (SD = 0.539). The mean lengths of the index (2D) and ring (4D) fingers were 58.49 mm (SD = 3.221) and 58.46 mm (SD = 2.533), respectively. The prevalence of the different molar relationships, based on Angle's classification of malocclusion, is presented in (Table 2, Figure 2). Class I malocclusion was the most prevalent, accounting for 73% of the sample, followed by Class II (18.5%) and Class III (8.5%). Regard the 2D:4D ratio, 53.2% of the participants had a ratio of 1 or more, while 46.8% had a ratio of less than 1 (Table 3). The relationship between the 2D:4D ratio and the different molar relationships were analyzed using the Chi-Square test. The results showed no statistically considerable relationship between the 2D:4D ratio and the molar relationship types (Class I, Class II and Class III), with a p-value of 0.904 (Table 4). The distribution of the 2D:4D ratio across the different malocclusion classes was nearly identical Figure 3. Of the participants with Class I malocclusion, 53.8% had a ratio of 1 and more, compared to 51.3% in the Class II group and 51.4% in the Class III group. The remaining participants had a ratio of <1, with 46.2% of Class I, 48.7% of Class II and 48.6% of Class III showing this ratio.

## Discussion:

In the field of dentistry, research exploring the impact of hormonal fingerprints on oral health remains limited, prompting efforts to investigate whether the 2D:4D ratio could serve as a predictor of an individual's risk for malocclusion [7]. The 2D:4D ratio refers to the relative lengths of the index (2nd) and ring (4th) fingers and it has been identified as a reliable marker of prenatal testosterone exposure. Higher levels of testosterone during fetal development are typically associated with a longer ring finger compared to the index finger, influencing the 2D:4D ratio [10]. Mandibular growth, suggesting that the 2D:4D ratio could serve as a non-invasive and reproducible marker for mandibular prognathism. This aligns with the idea that the 2D:4D ratio might be useful not only in predicting dental and craniofacial development but also as a diagnostic tool in understanding the hormonal influences on mandibular growth. Therefore, it exploring the connection between the 2D:4D ratio and malocclusion is of particular interest to researchers studying craniofacial growth patterns [11]. This research meant to explore the potential connection between the 2D:4D ratio and malocclusion types in a sample of male students aged 12 to 15 years from Alrass City, Saudi Arabia. The results specify that the 2D:4D ratio does not exhibit a statistically considerable association with the type of malocclusion in this population. Regarding the prevalence of malocclusion, Class I malocclusion was the most common, in line with other epidemiological studies that report Class I as the predominant malocclusion type in various populations. Class II and Class III malocclusions were observed less frequently, which is typical of the distribution seen in many age groups [12-13].

There has been significant interest in the 2D:4D ratios as a potential biomarker for predicting skeletal and dental traits with researchers evaluating the association of the hormonal fingerprint with craniofacial shape, cognition, dental caries and malocclusion. Valla *et al.* investigated 2D:4D ratio for its relation with craniofacial shape in prepubertal children and found no correlation [14]. Ramaneshwar *et al.* explored the association between hormonal fingerprint and examination grades as well as cognition but found no significant relationship of 2D:4D ratios with the tested cognitive domains of fluency, recall or memory [15]. Our analysis found no statistically considerable connection between the 2D:4D ratio and the different molar relationships classified by Angle. The Chi-Square test revealed a p-value of 0.904, suggesting that variations in the 2D:4D ratio was not related to the prevalence of Class I, II, or III malocclusion in this sample. The distribution of the 2D:4D ratios across the different malocclusion classes was nearly identical, with a slight majority of participants in each class having a ratio of 1 and the remaining participants displaying a ratio of less than 1.

While several previous studies have suggested a correlation between the 2D:4D ratio and various health and dental conditions, such as the study by Priyanka *et al.* which found a direct connection between the ratio and the rate of malocclusion, with statistical significance [16] and Garg *et al.* who reported a relationship between a high 2D:4D ratio and greater rates of malocclusion [17]. These results are not in conformity with our study. It is pertinent to note here that the study by Garg *et al.* had enrolled college students (age-group 18-25 years) and more importantly used the Dental esthetic index to record malocclusion. Hence a comparison with our study findings would be misleading. These findings are also not correlated with the results of Issrani *et al.* where no statistically considerable connection was found between the 2D:4D ratio and malocclusion status in their sample [18] Beegum *et al.* in their research concluded that the children with low 2D:4D ratio have higher caries scores and suggested its use as a biological predictor for dental caries [8]. This lack of correlation between 2D:4D Ratio and malocclusion in our research could also be due to differences in the sample population; the relatively small and homogeneous sample of male students from a single city may limit the generalizability of the findings, methodology, or other confounding factors not accounted for in this study.

## Conclusion:

The 2D:4D ratio is a useful tool for investigating prenatal hormonal influences. This data suggest that it does not serve as a reliable predictor of malocclusion. Hence, further research should consider larger, more diverse populations and investigate additional factors that might better explain the development of malocclusion, including genetic, environmental and hormonal influences during different stages of development.

## Figures and Tables

**Table 1:** Descriptive data of the study population

**Variable**	**Minimum**	**Maximum**	**Mean**	**Std. Deviation**
Age	12	15	13	0.539
Digit 2	53 mm	67 mm	58.49 mm	3.221 mm
Digit 4	55 mm	63 mm	58.46 mm	2.533 mm

**Table 2:** Prevalence of different molar relationships in the study sample

**Molar Relation**	**Number**	**Percentage %**
Class I	299	73%
Class II	76	18.50%
Class III	35	8.50%
Total	410	100.00%

**Table 3:** Prevalence of different molar relationships in the study sample

**2D:4D Ratio**	**Number**	**Percentage**
≥1	218	53.20%
<1	192	46.80%

**Table 4:** Relationship between 2D:4D ratio and molar relationship

**2D:4D Ratio**	**Molar Relation**								**χ^2^ (p)**
	**Class I (N=299)**		**Class II (N=76)**		**Class III (N=35)**		**Total (N=410)**		
	**No**	**%**	**No**	**%**	**No**	**%**	**No**	**%**	
≥1	161	53.80%	39	51.30%	18	51.40%	161	53.80%	0.202 (p=0.904)
<1	138	46.20%	37	48.70%	17	48.60%	138	46.20%	
